# Cationic Surfactants as Disinfectants against SARS-CoV-2

**DOI:** 10.3390/ijms23126645

**Published:** 2022-06-14

**Authors:** Eduard V. Karamov, Viktor F. Larichev, Galina V. Kornilaeva, Irina T. Fedyakina, Ali S. Turgiev, Andrey V. Shibaev, Vyacheslav S. Molchanov, Olga E. Philippova, Alexei R. Khokhlov

**Affiliations:** 1 Gamaleya National Research Center for Epidemiology and Microbiology of the Russian Ministry of Health, 123098 Moscow, Russia; karamov2004@yandex.ru (E.V.K.); vlaritchev@mail.ru (V.F.L.); kornilaeva@yandex.ru (G.V.K.); irfed2@mail.ru (I.T.F.); turgiev@ld.ru (A.S.T.); 2 National Medical Research Center of Phthisiopulmonology and Infectious Diseases of the Russian Ministry of Health, 127473 Moscow, Russia; 3 Physics Department, Moscow State University, 119991 Moscow, Russia; shibaev@polly.phys.msu.ru (A.V.S.); molchan@polly.phys.msu.ru (V.S.M.); khokhlov@polly.phys.msu.ru (A.R.K.)

**Keywords:** SARS-CoV-2, COVID-19, cationic surfactants, virucidal activity, quaternary ammonium compounds, disinfectants

## Abstract

The virucidal activity of a series of cationic surfactants differing in the length and number of hydrophobic tails (at the same hydrophilic head) and the structure of the hydrophilic head (at the same length of the hydrophobic n-alkyl tail) was compared. It was shown that an increase in the length and number of hydrophobic tails, as well as the presence of a benzene ring in the surfactant molecule, enhance the virucidal activity of the surfactant against SARS-CoV-2. This may be due to the more pronounced ability of such surfactants to penetrate and destroy the phospholipid membrane of the virus. Among the cationic surfactants studied, didodecyldimethylammonium bromide was shown to be the most efficient as a disinfectant, its 50% effective concentration (EC50) being equal to 0.016 mM. Two surfactants (didodecyldimethylammonium bromide and benzalkonium chloride) can deactivate SARS-CoV-2 in as little as 5 s.

## 1. Introduction

The ongoing coronavirus disease (COVID-19) pandemic caused by severe acute respiratory syndrome coronavirus 2 (SARS-CoV-2) is the most dramatic public health crisis in the last 100 years. By 30 May 2022, there were over 531 million infected people and 6.3 million deaths worldwide [1]. Therefore, it is extremely important to use multiple means of controlling the disease. Although airborne, and droplet transmission are the most common ways of SARS-CoV-2 spreading, contact transmission (through contaminated hands and surfaces touched by infected persons) also takes place. It was demonstrated that at room temperature SARS-CoV-2 survives for up to 3–4 days on plastic [2,3,4] and stainless-steel surfaces [2,3] and for up to 2 days-on glass and banknotes [3]. To avoid contact transmission, it is necessary to use hand sanitizers and efficiently disinfect surfaces, especially in public places like hospitals, transport, stores, malls, etc. At present, most of the hand sanitizers used for SARS-CoV-2 control are represented by alcohol-based solutions [5]. Some of them (containing alcohol at high concentrations) are flammable; also, they evaporate rapidly thereby shortening the exposure time; among other disadvantages is their ability to cause skin drying/cracking [6] and induce contact and atopic dermatitis [7]. As an alternative, one can consider another commonly used wide class of disinfectants—quaternary ammonium compounds (quats) [8,9] often called the “workhorses” of modern disinfection [10]. They are non-flammable and some of them (for example, benzalkonium chloride (BAC)) are less irritating to the skin [5]. Quaternary ammonium compounds exert antiviral effects by disrupting the lipid bilayer of viral envelopes [5,11] (all coronaviruses, including SARS-CoV-2, belong to enveloped viruses) [12]. The spike (S) of SARS-CoV-2, responsible for its entry, is an integral glycoprotein of the lipid membrane of the virions. After binding to its cellular receptor, S-protein undergoes conformational changes resulting in the fusion of the cellular and viral membranes. Virions with damaged lipid membranes cannot enter the cells and initiate the infection. Therefore, disruption of the virion membrane contributes to SARS-CoV-2 inactivation. Another mechanism of the antiviral activity of quaternary ammonium compounds consists in their lysosome tropism and accumulation in lysosomes or endosomes, ultimately blocking viral entry [11].

The most common quaternary ammonium disinfectants are probably BAC, cetylpyridinium chloride (C16Py), and didodecyldimethylammonium chloride/bromide [8]. BAC is utilized in hand sanitizers, soaps, cleaning wipes, hospital sanitation kits, surface disinfectants [5,10,11], etc. C16Py is widely used in mouthwash, toothpaste, cough lozenges, and so on [13,14]. The newer fourth-generation quaternary ammonium compounds, referred to as twin-chain or dialkyl quaternaries (e.g., didodecyldimethylammonium bromide (C12-C12DMA)), are used for the stabilization of emulsions [15]. They have some advantages since they remain active in hard water and are tolerant to anionic residues [16].

Even now, little is known about the resistance of SARS-CoV-2 to cationic surfactant-based common disinfectants. In particular, it was shown that a 0.2 wt% aqueous BAC solution reduces the infectious titer of SARS-CoV-2 by 3 orders of magnitude in 1 min [5,17]; in another paper [3], it was claimed that SARS-CoV-2 becomes undetectable after 5 min treatment with a less concentrated (0.1 wt%) BAC. As for the twin-chain cationic surfactant di-N-decyldimethylammonium chloride (C10-C10DMA), it was shown to reduce SARS-CoV-2 by almost 5 orders of magnitude at concentrations exceeding 283 mg/L (0.028 wt%) [18]. Moreover, virucidal activity against SARS-CoV-2 was found for the cationic surfactant C16Py [11,13] in vitro and for a mouthwash with 0.075 wt% C16Py (Colgate Plax) in vivo [14]. However, none of those studies compared the virucidal activities of the different cationic disinfectants against SARS-CoV-2.

**Figure 1:**
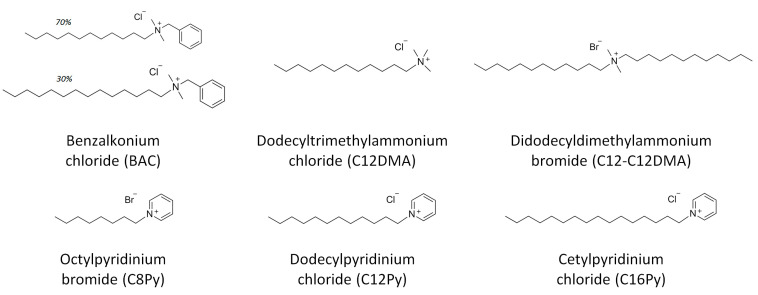
Molecular structure of cationic surfactants under study.

Therefore, the aim of the present work is to assess the virucidal efficacy against SARS-CoV-2 for diverse cationic surfactant-based disinfectants and to reveal the role of their structure (length and number of their hydrophobic tails and type of head) on the activity. For these studies, we selected the three most common disinfectants based on cationic surfactants (BAC, C16Py, and C12-C12DMA) as well as their analogs differing in the length and number of surfactant tails and the structure of hydrophilic head (as depicted in Figure 1). We believe that the results obtained will help prepare cationic disinfectants of optimum composition, e.g., for surfaces and hands contaminated with SARS-CoV-2.

## 2. Results and Discussion

### 2.1. Effect of the Surfactant Structure on the Virucidal Efficacy

Comparative studies of the virucidal activity were carried out at fixed surfactant concentration (0.28 mM) and contact time (1 h). For most of the surfactants (except C12-C12DMA) the concentration used was below the critical micelle concentration (cmc). The results are summarized in Table 1. They demonstrate that, under those conditions, four surfactants (C12BAC, BAC, C12-C12DMA, and C16Py) ensure complete inhibition of the infection (inhibition coefficient IC = 100%) and reduce SARS-CoV-2 titer by 7 orders of magnitude.

**Figure 2:**
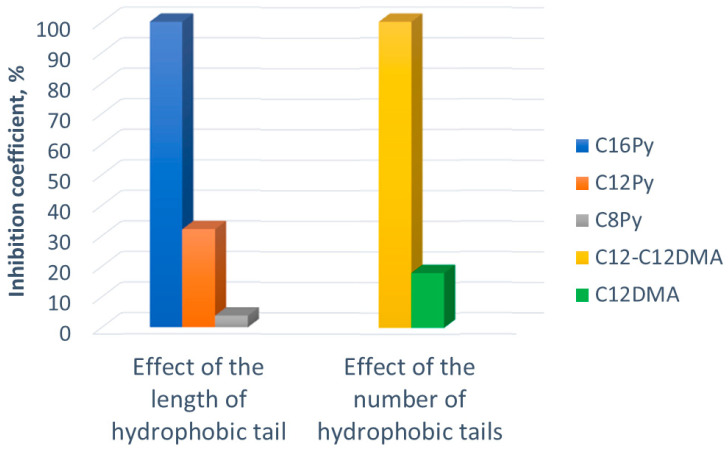
Effects of hydrophobicity on the virucidal activity of cationic surfactants against SARS-CoV-2: (**left**) effect of the length of the hydrophobic tail (for C8Py, C12Py, and C16Py surfactants having the same hydrophilic pyridinium head group) and (**right**) effect of the number of hydrophobic tails (for C12DMA and C12-C12DMA surfactants) on the values of the inhibition coefficient, observed at 0.28 mM concentration of each surfactant.

Structural determinants of the virucidal activity of the surfactants were studied. We first assessed the effect of the length of hydrophobic tails. For that, we compared activities of a series of N-alkylpyridinium surfactants (C16Py, C12Py, and C8Py), which have the same hydrophilic pyridinium head and hydrophobic n-alkyl tails of different lengths (Figure 1). As shown in Table 1 and Figure 2, increasing the length of the hydrophobic tail of a surfactant enhances its activity against SARS-CoV-2. Previously, C16Py was shown to destabilize the SARS-CoV-2 membrane through electrostatic interactions of the cationic head groups of the surfactant with the negatively charged viral membrane, as was detected by the shift in zeta potential [13]. Simultaneously, the hydrophobic groups of the surfactant penetrate the hydrophobic interior of the phospholipid bilayer of the viral membrane, thereby destroying it [32]. Higher surfactant hydrophobicity favors this process.

A comparison of the data obtained for alkylammonium surfactants C12-C12DMA (with two C12 tails) and dodecyltrimethylammonium chloride C12DMA (with one C12 tail), having similar ammonium head group (Figure 1), shows that the two-tailed surfactant is 5.5-fold more active against the virus (as judged by IC values; Table 1 and Figure 2). This finding lends support to the idea that surfactant hydrophobicity is a key factor in virucidal activity. Note that the change of the type of counterion from chloride to bromide does not affect the virucidal activity against SARS-CoV-2 as was clearly demonstrated for di-N-decyldimethylammonium chloride and bromide recently [18].

One can also compare the virucidal activity of C12BAC and C12DMA, which have the same hydrophobic tail (C12), but differ in the presence of an additional substituent (benzene ring) at the surfactant head (Figure 1). It follows from Table 1 that the introduction of this substituent causes a 5.5-fold increase in IC. This also appears to be underlain by enhanced hydrophobicity of the surfactant molecule.

The contribution of surfactant head type to the antiviral effects was further studied using two surfactants with the same hydrophobic tail (C12) and different heads, i.e., pyridinium chloride C12Py and trimethylammonium chloride C12DMA (Figure 1). The data in Table 1 demonstrate that C12Py exhibits a more pronounced virucidal activity. This effect can also be related to the higher hydrophobicity of the pyridinium chloride head, which contains more nonpolar groups as compared to the trimethylammonium head [30].

Now let us consider the correlations between the virucidal activity and such characteristics of surfactants as cmc and hydrophile-lipophile balance (HLB) values. The cmc value (i.e., the concentration at which surfactant molecules start to aggregate into micelles) is an important characteristic related to the free energy of micelle formation, ΔG_mic_, as [33,34]:

RT ln cmc = ΔG_mic_. (1)

**Figure 3:**
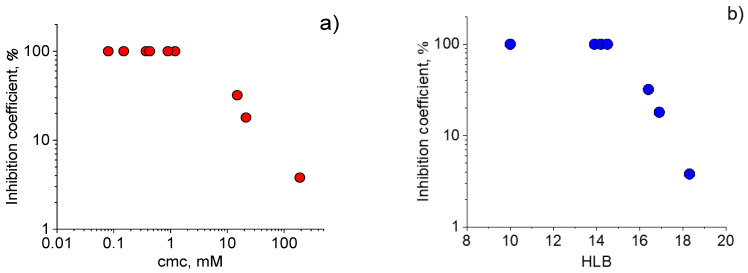
Inhibition coefficient as a function of: (**a**) critical micelle concentration cmc and (**b**) hydrophile-lipophile balance HLB of different cationic surfactants for SARS-CoV-2 inactivation by 0.28 mM surfactant solutions (contact time 1 h).

One can see from Figure 3a that the virucidal activity increases with decreasing cmc. Therefore, the surfactants more prone to aggregation possess better virucidal properties against SARS-CoV-2. This may be related to the fact that such surfactants are easily incorporated into the virion’s phospholipid bilayer.

Another important characteristic of surfactants is the HLB value - the number related to the hydrophilic to lipophilic moieties ratio. This value, connected to the work of surfactant transfer from water to oil phase [35], serves as an empirical measure of the relative hydrophobicity [36,37]: the lower the HLB, the stronger the hydrophobicity. Figure 3b shows that HLB values correlate with virucidal activity demonstrating that the activity increases with surfactant hydrophobicity. Note that Figure 3b presents the HLB values for C12DMA surfactant averaged over three values obtained in different papers (Table 1).

Thus, our study of the effect of the structure of cationic surfactants on the virucidal activity against SARS-CoV-2 showed that the introduction of additional hydrophobic groups into surfactants augments their activity. This may be due to the more pronounced capacity of such surfactants for penetrating and destroying the phospholipid membrane of SARS-CoV-2.

### 2.2. Concentration Dependence of Virucidal Activity of the Most Efficient Surfactants

Table 2 shows the effect of the concentration of cationic surfactants on SARS-CoV-2 inactivation at constant contact time (1 h). For those studies, the four most efficient surfactants (C12BAC, BAC, C12-C12DMA, and C16Py) were used. From Table 2 it is clear that the virucidal activity increases with increasing surfactant concentration. It appears that the density of surfactant ions acting on the lipid membrane is an important factor. At 0.0048 mM, even prolonged incubation (1 h) favoring a gradual influx of surfactant ions does not result in pronounced SARS-CoV-2 inactivation. Complete inactivation occurs at surfactant concentrations exceeding 0.112 mM.

The 50% effective concentrations (EC50) were derived from concentration dependences of virucidal activity (expressed as IC values). The double-chain surfactant C12-C12MA exhibits the lowest EC50 (0.016 mM; Table 2) and, therefore, maximum efficiency in inactivating SARS-CoV-2. This may be due to its highest hydrophobicity among the surfactants under study. In addition, the presence of two chains may facilitate the penetration of the viral membrane, thereby accounting for the stronger antiviral activity of double-chain surfactants. Indeed, the second hydrophobic group (lying some distance off the first one) likely augments the membrane-perturbing effect.

The values of EC50 for BAC and C12BAC are equal to 0.072 and 0.081 mM, respectively (Table 2). Those surfactants possess the same hydrophilic head but differ in the length of hydrophobic groups (C12BAC contains only C12 tails, whereas BAC has 70% of C12 tails and 30% of C14 tails). Thus, the lower EC50 for BAC correlates well with its higher hydrophobicity.

As regards C16Py, its EC50 value (0.101 mM; Table 2) exceeds those of other surfactants under study. Note that C16Py and C12BAC have the same number of carbon atoms contributing to hydrophobicity (21 carbon atoms), the same empirical formula (C21H38ClN), and close HLB values. Moreover, each of the two surfactants has hydrophobic fragments (a long n-alkyl chain and an aromatic ring) on either side of its cationic group (Figure 1). Nevertheless, C12BAC exhibits a higher activity against SARS-CoV-2. It is conceivable that the difference in the activity may be due to the presence in C12BAC of a CH_2_-group separating the aromatic ring from the cationic group. This additional group likens C12BAC to two-chain surfactants capable of perturbing the membrane more efficiently.

**Figure 4:**
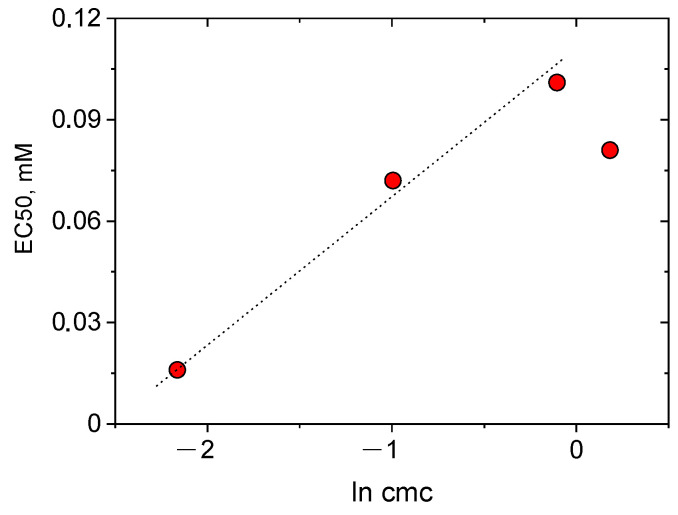
50% effective concentrations EC50 for SARS-CoV-2 inactivation as a function of critical micelle concentration cmc of different cationic surfactants in semi-logarithmic representation.

For all substances under study, their EC50s are 5-10-fold lower than their respective cmc values (Table 1 and Table 2), suggesting that the virucidal effects are produced by the nonaggregated surfactants. This implies that surfactant monomers (rather than aggregates) are interacting with the lipid structures, which is consistent with the results obtained for solubilization of phosphatidylcholine bilayers of liposomes by cationic alkyl pyridinium surfactants [38]. Figure 4 shows that the EC50 values decrease with decreasing cmc. Note that the three first points of the dependence of EC50 on the logarithm of cmc lie on the same line (Figure 4) suggesting that, in this range of cmc values, EC50 is directly proportional to the free energy of micelle formation, ΔG_mic,_ since ln cmc ~ ΔG_mic_ (Equation (1)).

From Table 1 and Table 2 it is evident that for all surfactants the EC50 values decrease with HLB, that is with increasing hydrophobicity. This is related to the enhanced ability of the surfactants to interact with lipophilic lipid bilayers of the virus.

Thus, among the studied surfactants, C12-C12DMA has the lowest EC50 value (0.016 mM). It may be accounted for by its double-chain structure in addition to the overall greater hydrophobicity.

### 2.3. Time Dependence of Virucidal Activity of the Most Efficient Surfactants

**Figure 5:**
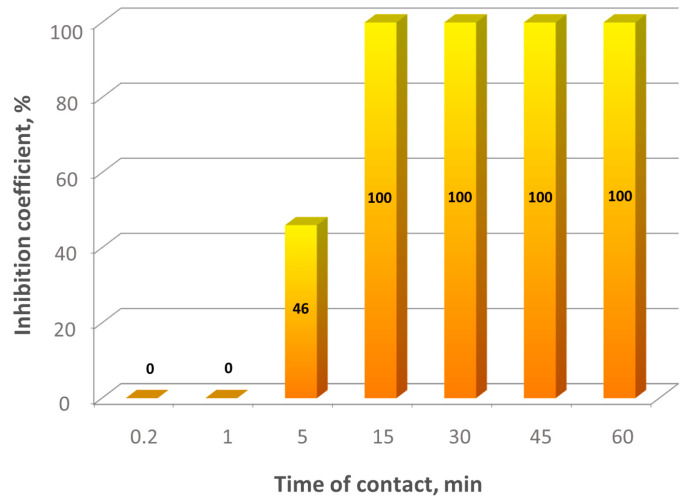
Kinetics of SARS-CoV-2 inactivation by 0.14 mM benzalkonium chloride at different virus-disinfectant contact times.

Figure 5 demonstrates time dependence of SARS-CoV-2 inactivation by 0.14 mM (0.005 wt%) BAC. It is evident that BAC starts to inactivate the virus after a 5-min contact and completes the inactivation in 15 min. It can be assumed that, for faster inactivation, higher surfactant concentrations are needed.

Therefore, subsequent experiments were performed at 20-fold higher surfactant concentrations, 2.8 mM. For those studies, we used three cationic surfactants, BAC, C16Py, and C12-C12DMA. The results are summarized in Table 3. It is seen that two surfactants (BAC and C12-C12DMA) completely inactivate SARS-CoV-2 at contact times as short as 5 s. The efficiency of BAC against SARS-CoV-2 was reported previously in several papers [3,5,17]. For instance, a 0.1 wt% aqueous solution of BAC was shown to inactivate the virus after 5 min of treatment [3]. In the present study, we demonstrate that a 5-s incubation with 0.1 wt% solution of this surfactant is sufficient to ensure complete SARS-CoV-2 inactivation (Table 3). These results also indicate that BAC efficiency is much higher than found in another report [17], where SARS-CoV-2 treatment with 0.2 wt% BAC for 5 s led to as little as 1.83 log reduction of the virus titer. As regards C12-C12DMA, the data obtained are consistent with those reported for another two-chain cationic surfactant, di-N-decyldimethylammonium chloride [18]; in the present study, however, a much shorter contact time was sufficient (5 s instead of 30 s).

In addition, Table 3 shows that 2.8 mM C16Py inactivates SARS-CoV-2 completely after no less than 5 min of contact. This is consistent with the weaker antiviral activity of this surfactant, evidenced by its higher EC50 value (as compared to those of the other two surfactants; Table 2).

Thus, BAC and C12-C12DMA, which ensure SARS-CoV-2 inactivation in as little as 5 s of contact, are the most promising disinfectants among the surfactants under study.

## 3. Materials and Methods

### 3.1. Surfactants

Benzyldimethyldodecylammonium chloride C12BAC (>99%) from Sigma Aldrich (Saint Louis, MO, USA, product number 13380), benzalkonium chloride BAC (>95%) containing 70% C12BAC and 30% benzyldimethyltetradecylammonium chloride from Sigma Aldrich (product number 12060), didodecyldimethylammonium bromide C12-C12DMA (>98%) from ABCR (Karlsruhe, Germany), cetylpyridinium chloride C16Py (>98%) from Sigma Aldrich, dodecylpyridinium chloride C12Py (>99%) from ABCR, octylpyridinium bromide C8Py (>99%) from Chemos GMbH (Altdorf, Germany), dodecyltrimethylammonium chloride C12DMA (>97%) from ABCR, pyrene for fluorescence (>99%) from Sigma Aldrich were used as received. All solutions were prepared by weighing with dissolving the surfactant in physiological solution (0.9 wt% NaCl) as a solvent. Distilled deionized water for the preparation of the solutions was obtained using an ultrapure water purification system Milli Q (Millipore, Burlington, MA, USA).

### 3.2. Cells

Vero E6 cells (ATCC, Manassas, VA, USA; catalog number CRL-1586), a continuous line isolated from African green monkey (*Chlorocebus* sp.) kidney epithelium, were cultured in high glucose Dulbecco’s modified Eagle’s medium (DMEM; Sigma-Aldrich, St. Louis, MO, USA) supplemented with 5% fetal calf serum (FCS), 2 mM L-glutamine, 150 u/mL penicillin, and 150 u/mL streptomycin (all from Thermo Fisher Scientific, Waltham, MA, USA) (growth medium) at 37 °C in 5% CO_2_. This lineage is widely documented to be sensible and permissive to SARS-CoV-2 infection, leading to high titer replication [39].

### 3.3. Virus and Virus Titration

SARS-CoV-2 used in this work was a clinical isolate (hCoV-19/Russia/Moscow-PMVL-12/2020; GISAID reference EPI_ISL_572398) belonging to B.1.1.4 lineage [40]. The viral stock was propagated in confluent Vero E6 monolayers, harvested on day 5, concentrated by centrifugation at 140,000× *g* and 4 °C for 1 h (Optima XPN 100, Beckman Coulter, Brea, CA, USA) to achieve 1 × 10^8^ median tissue culture infectious doses (TCID_50_) per 1 mL, and stored at −80 °C.

TCID_50_ is the measure of infectious virus titer; this endpoint dilution assay quantifies the amount of virus required to produce cytopathic effects (CPE; structural changes in host cells, caused by the viral invasion and leading to cell death) in 50% of inoculated tissue cultures. Determination of TCID_50_ is one of the established methods of SARS-CoV-2 quantification [41]; 10 TCID_50_ were shown to be equivalent to 2–4 infectious virions [42,43], which is somewhat less than the theoretical value (equal to 7 [44]).

**Figure 6:**
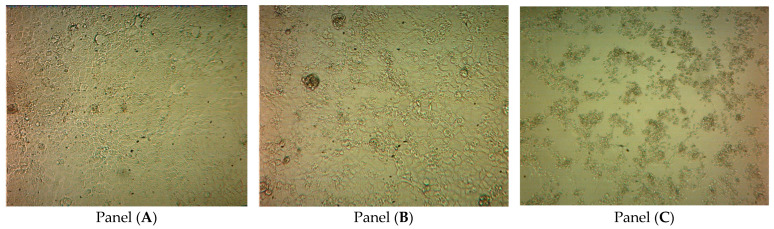
Micrographs displaying the time course of the development of SARS-CoV-2-induced cytopathic effects CPE in the Vero E6 cell monolayer. The CPE were visually scored for each well in a blinded fashion by two independent observers. Wells with 0, 25, 50, 75, and 100% cells exhibiting CPE or viability loss were scored, respectively, CPE–, CPE+, CPE++, CPE+++, and CPE++++. The photo in Panel (**A**) shows non-infected cells (no-virus control) after 72 h of incubation (no CPE or dead cells). Panels (**B**) and (**C**) show, respectively, cells inoculated with 10^3^ TCID_50_/mL 36 and 72 h post-infection. About 50% of cells in *Panel B* exhibit CPE or loss of viability (score: CPE++). In Panel (**C**), all cells are dead (score: CPE++++). The photos were taken using an inverted microscope (×200 magnification; Leitz Diavert, Wetzlar, Germany).

In brief, a suspension of Vero E6 cells in a growth medium (1.2 × 10^6^ cells/mL) was introduced in 96-well flat-bottomed Costar tissue culture plates (Corning, Corning, NY, USA) at 100 µL/well and cultured at 37 °C in 5% CO_2_ for 24 h (until the formation of confluent monolayers). Thereafter, the monolayers were washed with FCS-free DMEM (2 × 5 min) and inoculated with serial 10-fold dilutions of the virus (10^1^–10^8^ TCID_50_/mL) in a support medium (DMEM, 1% FCS) at 100 µL/well. Each dilution was tested in eight replicates; in every plate, eight wells were used as no-virus control. Following a 2-h incubation (at 37 °C in 5% CO_2_) for virus adsorption, the inoculum was removed, and the plates were washed twice with FCS-free DMEM, filled with (DMEM, 2% FCS), and further incubated (at 37 °C in 5% CO_2_) for 96 h. The plates were observed daily to monitor the development of virus-induced CPE, which was completed in 72–96 h (Figure 6).

To confirm the results of visual observation, cell viability was further assessed by the MTS test (CellTiter 96^®^ AQ_ueous_ One Solution Cell Proliferation Assay; Promega, Madison, MI, USA; catalog number G3582) based on the ability of live cells to convert 3-(4,5-dimethylthiazol-2-yl)-5-(3-carboxymethoxyphenyl)-2-(4-sulfophenyl)-2H-tetrazolium, inner salt (MTS) into a colored formazan product that is soluble in tissue culture medium (this conversion, presumably accomplished by NADPH or NADH produced by dehydrogenase enzymes in metabolically active cells [45,46], is to a certain extent directly proportional to the concentration of viable cells). When the incubation was completed, the culture medium was removed from the wells, and 100 µL of support medium (DMEM, 2% FCS) and 20 µL MTS reagent were added to each well, and the plate was incubated at 37 °C for additional 3 h. Absorbance was measured at 490 nm on an iMark plate reader (Bio-Rad Laboratories, Hercules, CA, USA) using 630 nm as a reference wavelength. No discrepancies between the two methods of CPE assessment have been observed. The percentages of cultures with virus-induced CPE or viability loss were recorded for each virus dilution; the titer was calculated using the Spearman–Kärber method and presented as TCID_50_/0.1 mL or lg TCID_50_/0.1 mL [47,48].

### 3.4. Fluorescence Spectroscopy

**Figure 7:**
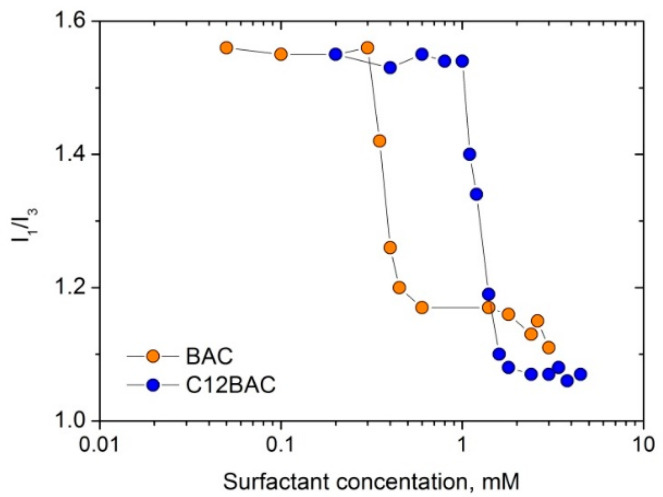
Polarity parameter of pyrene I_1_/I_3_ as a function of the concentration of cationic surfactants: benzalkonium chloride BAC and benzyldimethyldodecylammonium chloride C12BAC.

Fluorescence spectroscopy measurements with pyrene as a probe were performed with the Perkin Elmer LS-55 spectrofluorimeter. The excitation wavelength was 338 nm, and 7 and 3 nm spectral slits were used for excitation and emission, respectively. Samples for measurements were prepared by first pipetting 0.01 mL of pyrene stock solution (10^−4^ M in ethanol) into a flask, and evaporating ethanol at ambient conditions. Then, 1 mL of a surfactant solution of a given concentration was added to the flask and stirred for 1 day before the measurements. The ratio of the first (371 nm) to the third (383 nm) vibronic peaks in fluorescence spectra of pyrene I_1_/I_3_ is known to be sensitive to the polarity of its microenvironment [49,50]. The formation of micelles and the penetration of hydrophobic pyrene molecules in their cores leads to the drop in the polarity parameter I_1_/I_3_ (Figure 7). The cmc was determined as an inflection point of the dependence of I_1_/I_3_ on surfactant concentration. The cmc of the surfactants are displayed in Table 1.

### 3.5. Evaluation of Virucidal Activity

Virucidal activity is defined as the ability to kill viruses [51], i.e., to cause them to lose “viability”. In its turn, the “viability” of a virus is equivalent to its capacity for replication [52,53]; if the replication competence of a virus is irreversibly disrupted, the virus is no longer “alive”. To assess the virucidal activity of surfactants under study, we compared the replicability of surfactant- and mock-treated SARS-CoV-2 virions in permissive cells.

Surfactant solutions of a given concentration (0.0096 mM, 0.0448 mM, 0.224 mM, 0.56 mM, 1.12 mM, or 5.6 mM; in a volume of 1 mL) were incubated with an equal volume of the virus stock (10^8^ TCID_50_/_mL_) at room temperature for a certain period (contact time: 5 s, 10 s, 15 s, 30 s, 1 min, 5 min, 15 min, 30 min, 45 min, 60 min). To avoid the presence of surfactant during infection and its toxic effects on the cells, the samples (surfactant + virus) were centrifuged at 140,000× *g* (Optima XPN 100, Beckman Coulter, Brea, CA, USA) for 1 h. A positive control (the virus stock without surfactants) was used in every run. Viral pellets were resuspended in 300 µL of support medium (DMEM, 1% FCS), and, for each pellet, 10-fold dilutions in support medium were prepared. The titer of infectious SARS-CoV-2 was determined as described in Section 3.3 above. This endpoint dilution assay measures the amount of replication-competent SARS-CoV-2 particles directly: by the extent, to which the infection they induce is pronounced.

The virucidal efficacy of surfactants was assessed by the difference in the virus titers (A) between control (A_c_) and experimental (A_e_) samples:

A = A_c_ − A_e_

The protection index, or inhibition coefficient IC, was calculated for all concentrations and contact times using the formula:

IC = [(A_c_ − A_e_)/A_c_] × 100%

The values of the 50% effective concentrations EC50 were derived from IC dependences on surfactant concentrations, using non-linear regression analysis (Prism 6; GraphPad Software, San Diego, CA, USA).

## 4. Conclusions

In the present paper, we report on establishing a relationship between the molecular structure of the “workhorses” of modern disinfection [10], cationic surfactants, and their virucidal efficacy against SARS-CoV-2. It was shown that increasing the overall hydrophobicity and/or the number of hydrophobic fragments attached to the cationic head group of a surfactant enhances its virucidal activity. Those structural features presumably facilitate the incorporation of the surfactant into the lipid membrane of the virus and its subsequent disintegration.

Among the cationic surfactants studied, didodecyldimethylammonium bromide was the most effective. It has the lowest 50% effective concentration (EC50) − 0.016 mM (7.4 × 10^−4^ wt%). Didodecyldimethylammonium bromide, as well as benzalkonium chloride, were demonstrated to ensure fast (in 5 s) inactivation of SARS-CoV-2 at a surfactant concentration of 2.8 mM. Those surfactants may serve as a base of highly efficient disinfectants for hands and surfaces, which will limit the spread of COVID-19.

## Figures and Tables

**Table 1:** SARS-CoV-2 inactivation by 0.28 mM solutions of different cationic surfactants (contact time 1 h).

Surfactant	Abbreviation	Hydrophobic Tail	Critical Micelle Concentration (cmc), mM	Hydrophile-Lipophile Balance (HLB)	Virus Titer			Inhibition Coefficient IC, %
					Control A_c_	Experiment A_e_	Log10 Reduction A	
Benzyldimethyldode-cylammonium chloride	C12BAC	C12	1.2 *	14.2 **	7.00	0.00	7.00	100
Benzalkonium chloride	BAC	C12 (70%), C14 (30%)	0.37 *, 0.43 [19]	13.9 **	7.00	0.00	7.00	100
Didodecyldimethyl-ammonium bromide	C12-C12DMA	C12-C12	0.08 [20], 0.15 [21]	10 [22]	7.00	0.00	7.00	100
Dodecyltrimethyl-ammonium chloride	C12DMA	C12	21.3 [23]	15 [22], 17.1 [24], 18.5 [25]	7.00	5.75	1.25	18
Cetylpyridinium chloride	C16Py	C16	0.9 [26,27,28]	14.5 **	7.00	0.00	7.00	100
Dodecylpyridinium chloride	C12Py	C12	15 [29]	16.4 [30]	7.00	4.75	2.25	32
Octylpyridinium bromide	C8Py	C8	190 [29]	18.3 **	6.50	6.25	0.25	3.8

* in 0.9 wt% NaCl. ** calculated by group contribution method according to Refs. [30,31].

**Table 2:** SARS-CoV-2 inactivation by cationic surfactants of different concentrations (contact time 1 h).

Surfactant	Concentration		Virus Titer			Inhibition Coefficient IC, %
	mM	wt%	Control A_c_	Experiment A_e_	Log10 Reduction A	
Benzyldimethyldodecylammo- nium chloride (C12BAC)	0.0224	0.0008	6.5	5.5	1.0	15
	0.112	0.0038	6.5	2.0	4.5	69
	0.56	0.0190	6.5	0	6.5	100
	2.8	0.0950	6.5	0	6.5	100
	50% effective concentration (EC50) 0.081 mM					
Benzalkonium chloride (BAC)	0.0048	0.0002	6.5	6.0	0.5	7.7
	0.0224	0.0008	6.5	5.5	1.0	15
	0.112	0.0039	6.5	0	6.5	100
	0.56	0.0195	6.5	0	6.5	100
	2.8	0.0970	6.5	0	6.5	100
	50% effective concentration (EC50) 0.072 mM					
Didodecyldimethylammonium bromide (C12-C12DMA)	0.0048	0.0002	6.75	6.5	0.25	3.7
	0.0224	0.0010	6.75	2.0	4.75	70
	0.112	0.0052	6.75	0	6.75	100
	0.56	0.0259	6.75	0	6.75	100
	2.8	0.1295	6.75	0	6.75	100
	50% effective concentration (EC50) 0.016 mM					
Cetylpyridinium chloride (C16Py)	0.0048	0.0002	6.75	6.5	0.25	3.7
	0.0224	0.0008	6.75	6.0	0.75	11
	0.112	0.0038	6.75	0	6.75	100
	0.56	0.0190	6.75	0	6.75	100
	2.8	0.0950	6.75	0	6.75	100
	50% effective concentration (EC50) 0.101 mM					

**Table 3:** SARS-CoV-2 inactivation by 2.8 mM cationic surfactants at different virus-disinfectant contact times.

Surfactant	Contact Time	Virus Titer			Inhibition Coefficient IC, %
		Control A_c_	Experiment A_e_	Log10 Reduction A	
Benzalkonium chloride (BAC)	5 s	7.50	0.00	7.50	100
	15 s	7.50	0.00	7.50	100
	30 s	7.50	0.00	7.50	100
	5 min	7.50	0.00	7.50	100
Didodecyldimethylammonium bromide (C12-C12DMA)	5 s	8.00	0.00	8.00	100
	15 s	8.00	0.00	8.00	100
	30 s	8.00	0.00	8.00	100
	5 min	8.00	0.00	8.00	100
Cetylpyridinium chloride (C16Py)	5 s	7.75	6.00	1.75	23
	15 s	7.75	4.50	3.25	42
	30 s	7.75	3.25	4.25	55
	5 min	7.75	0.00	7.75	100

## Data Availability

Not applicable.
